# Willin/FRMD6: A Multi-Functional Neuronal Protein Associated with Alzheimer’s Disease

**DOI:** 10.3390/cells10113024

**Published:** 2021-11-04

**Authors:** Doris Chen, Wanjia Yu, Laura Aitken, Frank Gunn-Moore

**Affiliations:** School of Biology, University of St. Andrews, St. Andrews KY16 9TF, UK; dc242@st-andrews.ac.uk (D.C.); wy20@st-andrews.ac.uk (W.Y.); la49@st-andrews.ac.uk (L.A.)

**Keywords:** Willin/FRMD6, Hippo signaling, Alzheimer’s disease, neurodegeneration, mechanotransduction, oxidative stress

## Abstract

The FERM domain-containing protein 6 (FRMD6), also known as Willin, is an upstream regulator of Hippo signaling that has recently been shown to modulate actin cytoskeleton dynamics and mechanical phenotype of neuronal cells through ERK signaling. Physiological functions of Willin/FRMD6 in the nervous system include neuronal differentiation, myelination, nerve injury repair, and vesicle exocytosis. The newly established neuronal role of Willin/FRMD6 is of particular interest given the mounting evidence suggesting a role for Willin/FRMD6 in Alzheimer’s disease (AD), including a series of genome wide association studies that position Willin/FRMD6 as a novel AD risk gene. Here we describe recent findings regarding the role of Willin/FRMD6 in the nervous system and its actions in cellular perturbations related to the pathogenesis of AD.

## 1. The Willin/FRMD6 Protein

The 4.1-ezrin-radixin-moesin (FERM) domain-containing protein 6 (FRMD6), also known as Willin, is a ~71 kDa member of the 4.1 band protein superfamily which was first discovered as a novel binding partner of neurofascin 155 in rat sciatic nerves [1]. The human FRMD6 gene is found on the positive strand of chromosome 14 (cytogenic location 14q22.1) and contains 13 exons (https://gnomad.broadinstitute.org/gene/ENSG00000139926, accessed on 1 August 2021). Notably, the FRMD6 gene is in the same genomic region as FERMT2, an identified AD risk gene that is present in synapses and that interacts with amyloid precursor protein (APP), and SAV1, a Hippo signaling component [2].

**Figure 1:**
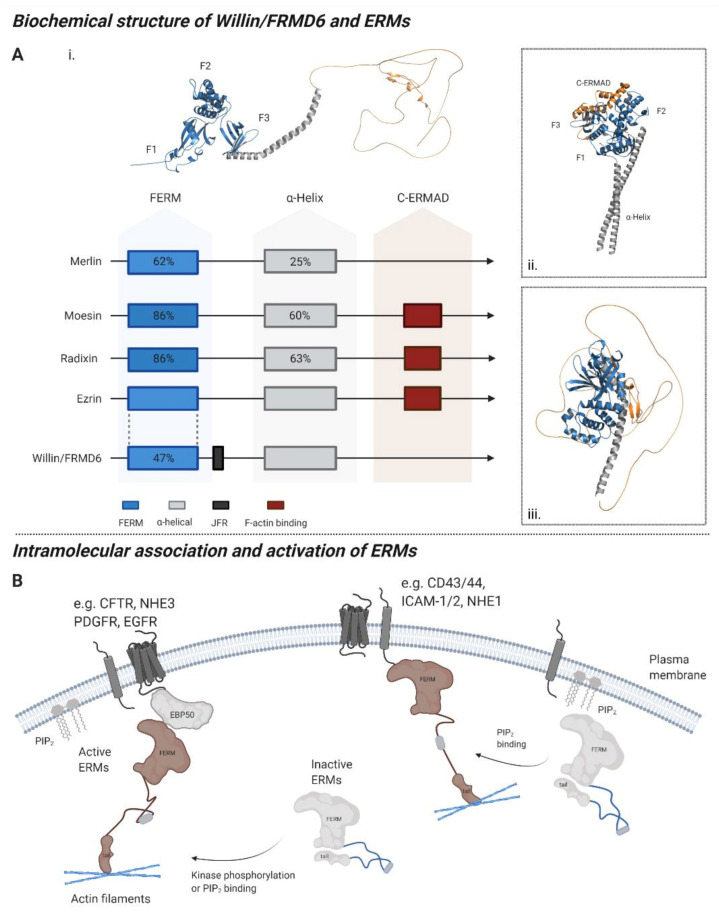
The biochemical structures and intramolecular associations of ERMs. Willin/FRMD6, ERMs, and Merlin share a similar biochemical structure and putative activation mechanism. (**A**) Biochemical structure of Willin/FRMD6 and ERMs: ERMs show very high sequence identity. Moesin and Radixin share 86% similarity with Ezrin in the FERM domain whereas Merlin is more divergent. The FERM domain of Willin/FRMD6 shares 47% protein similarity with Ezrin; however, Willin/FRMD6 lacks a filamentous actin (F-actin) binding domain. (i) Protein domain organization and sequence homology of the FERM domain containing proteins with the putative structure of active Willin/FRMD6. (ii) Crystal structure of full-length inactive Moesin (PDB ID: 2I1K [17]): the amino-terminal FERM domain (blue) consists of three subdomains: F1, F2, and F3. The central residue region (grey) is an α-helical rich coiled-coil. The carboxy-terminal ERM-associated domain (C-ERMAD, orange) contains the F-actin binding site. Intramolecular interactions between the FERM domain and C-ERMAD mask the F-actin binding domain. (iii) AlphaFold predicted structure of Willin/FRMD6 [18] showing the 3-lobed FERM domain and the central alpha helix. Significant disorder is present in the carboxy-terminal. (**B**) Intramolecular association and activation of ERMs: A conceptual model of the activation of ERMs involves the dissociation of the C-ERMAD from the FERM domain, thereby allowing the central α-helical region to unravel and free the F-actin and membrane protein binding domains. Activated ERMs can participate in microfilament–membrane linkage by direct association with transmembrane proteins. ERMs are activated through binding with PIP_2_ or phosphorylation at the Thr567 site. This unmasks the binding sites for specific membrane proteins such as CD43, CD44, ICAM-1/2, NHE1. Furthermore, EBP50 can also bind with the FERM domain. The PDZ domain of EBP50 can bind with additional proteins, such as CFTR, NHE3, PDGFR, β2 adrenergic receptor, and EGFR. Abbreviations: PIP_2_, Phosphatidylinositol 4,5-bisphosphate; ICAM1/2, Intercellular adhesion molecule 1/2; NHE1/3, Na+–H+ exchanger 1/3; EBP50, Adaptor protein ERM-binding phosphoprotein 50; CFTR, Cystic fibrosis transmembrane conductance regulator; EGFR, Epidermal growth factor receptor; JFR, a region adjacent to the FERM domain, termed the juxta-FERM domain region; PDGFR, Platelet-derived growth factor receptor. Created with BioRender.com, accessed on 1 August 2021.

Willin/FRMD6 contains an N-terminus FERM domain (residues 14–322) [1] and a juxta-FERM domain region (JFR) (residues 330–440) [3]. FERM domains are hydrophobic cysteine-rich regions of approximately 300 amino acids located near the N-terminus of proteins which assume a three-lobed cloverleaf structure [4,5,6,7]. FERM domains are unusual in their ability to bind both proteins and phospholipids; thus, these domains mediate interactions with the cytoplasmic tails of transmembrane proteins and with membrane-associated lipids [8,9]. While the FERM domain in Willin/FRMD6 shares only 47% sequence homology with that of characteristic FERM domain containing proteins ezrin, radixin, and moesin, collectively termed ERM proteins (Figure 1A), it is nevertheless capable of phospholipid binding [10]. Additionally, Willin/FRMD6 is also able to co-localize with the actin cytoskeleton though it lacks the C-terminal F-actin interaction motif of other ERM proteins [1]. The molecular structures of ERM proteins and Willin/FRMD6 as well as the putative mechanism for ERM protein activation are summarized in Figure 1. Independent of its FERM domain, Willin/FRMD6 interacts with apical polarity proteins Par3 and aPKC through its JFR domain, leading to its recruitment to adherens junctions [3]. Though Willin/FRMD6 lacks a kinase domain, it may be able to indirectly affect the phosphorylation state of downstream targets via interaction with other kinases [3]. 

Whether Willin/FRMD6 activation occurs through phosphorylation-driven unmasking of FERM domain residues (Figure 1B) remains to be established; however, high-throughput phosphor-proteomics studies have revealed several potential phosphorylation sites in Willin/FRMD6, including several within the FERM domain region such as S184 [11]. Phosphorylation at Y412 has been shown to occur in response to ephrin B1 activation of receptor tyrosine kinases [12]. Ischemia induces phosphorylation at S375 and S525 and MG132-modulation of proteasome activity induces phosphorylation at S544 [12].

In the neuroscience field, genome wide association studies (GWAS) have been used to identify candidate genes that are involved in Alzheimer’s disease (AD) risk and that thus present promising avenues for research and future therapeutic intervention. Recent GWAS and AD imaging studies have suggested Willin/FRMD6 as a novel gene involved in AD risk [13,14,15,16], but its potential function in the nervous system remains relatively unexplored and its role in neurodegeneration has yet to be established. In this review we discuss new findings regarding the functional role of Willin/FRMD6 in neuronal cells and highlight possible mechanisms through which Willin/FRMD6 dysfunction may contribute to AD pathogenesis. Specifically, we will address the following questions:

1. What is the expression pattern of Willin/FRMD6 in neurons and the nervous system?
2. What are the physiological functions of Willin/FRMD6?
3. Is Willin/FRMD6 dysregulated in AD?
4. If so, which mechanisms may explain its dysregulation?
5. What would be the functional consequences AD-induced Willin/FRMD6 dysregulation?

## 2. Expression of Willin/FRMD6

Willin/FRMD6 is widely distributed in various tissues and cell types including epithelial cells within the uterus, placenta, cervix, brain, heart, liver, prostate, and lungs [1,19,20,21]; fibroblasts of the peripheral nerves [1,21]; neurons of the peripheral (PNS) and central nervous systems (CNS) [22,23].

### 2.1. Regulation of Willin/FRMD6 Expression

Recent studies in cultured cell lines have revealed several mechanisms of Willin/FRMD6 transcriptional and post-transcriptional regulation. Metastasis Associated 1 Family Member 2 (MTA2) acts as a transcriptional repressor by binding to the promoter region of Willin/FRMD6 in hepatocellular carcinoma [24]. In cervical cancer cells, microRNA miR-454-3p inhibits Willin/FRMD6 protein expression by binding to Willin/FRMD6 mRNA [25]. In uterine corpus endometrial cancer, antisense long noncoding RNA FRMD6-AS2 enhances transcription of Willin/FRMD6 by binding to a distal regulatory element that facilitates DNA loop formation at the promoter region of Willin/FRMD6 [26]. Metformin, a well-known drug for the treatment of hyperglycemia [27,28], increases Willin/FRMD6 levels in Tamoxifen and paclitaxel sensitive breast cancer cells [29], suggesting that Willin/FRMD6 modulation may occur in response to glucose. Proteins that regulate Willin/FRMD6 expression include brain derived neurotrophic factor (BDNF) and cell polarity protein Crb3. BDNF-treatment of aged cardiac microvascular endothelial cells CMECs results in increased expression and transcription of Willin/FRMD6 [30]. In MCF10A cells, Crb3 knockdown results in decreased Willin/FRMD6 protein and mRNA expression, while Crb3 overexpression has the opposite effect [31]. Whether these regulatory mechanisms occur in neuronal cells presents a promising avenue for research. As discussed below, Willin/FRMD6 has neuronal-specific expression patterns and function. 

### 2.2. Willin/FRMD6 Sub-Cellular Expression in Mitotic Cells

In mitotic cells, Willin/FRMD6 expression has been observed in the cytoplasm, plasma membrane, cell nucleus, and cell–cell junctions [1,17,20,30,31]. Normal human oral mucosa cells display general cytoplasmic Willin/FRMD6 staining [1,19]. As has been hypothesized for other ERM proteins, this cytoplasmic pool of Willin/FRMD6 may be inactive, with activation occurring through phosphorylation events that allow binding to transmembrane and membrane-associated proteins [32,33]. Localization of Willin/FRMD6 to adherens junctions in fibroblasts is mediated by cell adhesion molecule nectin-1 [34]. In neuronal cell lines, Willin/FRMD6 staining at the cell periphery is typically granular or vesicular in nature [22], with no staining of plasma membrane protrusions as seen with other ERM proteins [32]. Localization of Willin/FRMD6 to the plasma membrane occurs in human squamous cell carcinomas [1,19] and in vitro with epidermal growth factor stimulation [1] and with cell-to-cell contact in confluent monolayers of PC12, HEK293, and SH-SY-5Y cells [1,35]. Nuclear localization of Willin/FRMD6 is observed in squamous cell carcinomas of the head, neck, and upper aerodigestive tract [19]. 14-3-3 proteins may mediate Willin/FRMD6 cytoplasmic retention as disruption of this interaction by point mutation of T28 to A in MCF7 breast cancer cells results in Willin/FRMD6 nuclear localization; however, it should be noted that these studies were conducted using a truncated form of Willin/FRMD6 and with an anti-FLAG antibody as a proxy for Willin/FRMD6 staining [36].

The functional implications behind the differing subcellular distributions of Willin/FRMD6 under both physiological and pathological processes have yet to be fully elucidated and what processes regulate Willin/FRMD6 subcellular localization are unknown. However, Willin/FRMD6 expression and function may be influenced by cellular aging as nerve fibers from older rats exhibit less intense staining compared to younger animals [22]. Consistently, staining is more intense in lower passage cell lines compared to older, more confluent passages [22]. Furthermore, migration of aging CMECs is driven by signaling via BDNF-TrkB-T1-Willin/FRMD6-Hippo, a modality which is not active in younger CMECs [30].

### 2.3. Willin/FRMD6 Expression in the Nervous System

**Figure 2:**
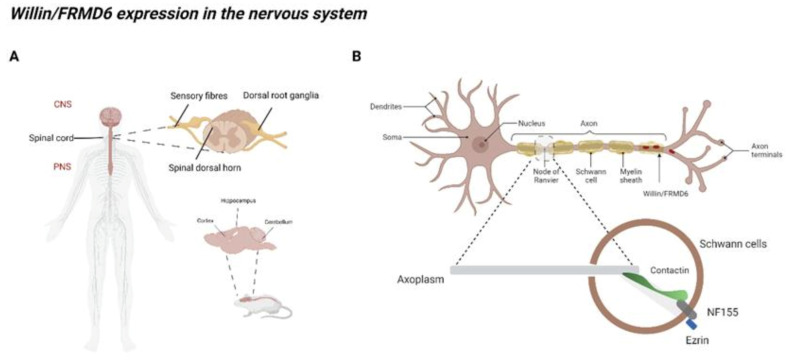
Willin/FRMD6 expression in the nervous system. Endogenous Willin/FRMD6 expression has been shown in various human and rodent tissues within and outside the nervous system. (**A**) Willin/FRMD6 protein has so far been found in both the CNS and PNS of humans. Within the PNS, Willin/FRMD6 is found in the spinal cord, the dorsal root ganglia, the spinal dorsal horn, and sensory fibres. Expression of Willin/FRMD6 has been shown in the cortex, hippocampus, and cerebellum of mouse brains; (**B**) Willin/FRMD6 was first identified in the rat sciatic nerve. FERM domain containing protein Ezrin binds to NF155 at the paranodal axoglial junction. NF155 interacts with the paranodal loops of myelinating glia to neuronal surface-associated proteins such as Contactin. Created with BioRender.com, accessed on 1 August 2021.

Recent studies have provided an insight into the endogenous localization of Willin/FRMD6 in the peripheral nervous system (PNS) and central nervous system (CNS) (Figure 2). In the PNS, Willin/FRMD6 expression is observed in fibroblasts and Schwann cells of rat sciatic nerves [21,23] and in the Schwann cell microvilli of myelinated mouse peripheral nerves [37]. 

Within neurons of the PNS, Willin/FRMD6 transcripts are found in the human and mouse sensory ganglia [38,39,40,41]. Furthermore, Willin/FRMD6 immunoreactivity is observed in the sensory ganglia corresponding to cranial nerves V, IX, and X of rat, xenopus, and humans [22] and in dorsal root ganglia (DRG), motor neurons, and the axons of the sciatic nerves in mice [23]. The distribution of Willin/FRMD6 in the periphery is non-homogeneous as it appears to be preferentially expressed in small and medium-sized DRG neurons, many of which are nociceptors [23] and is exclusively found in small caliber peptidergic fibers of cranial nerves containing both sensory and motor fibers (cranial nerves V, IX, and X) in rats, xenopus, and humans, with the highest immunoreactivity in cranial nerve X [22]. Willin/FRMD6′s widespread distribution in the nerves and ganglia indicates that it may have functional significance in multiple sensory modules and in motor control.

In the CNS, Willin/FRMD6 transcripts are found in the human and mouse spinal cord [38,39,40,41] and ubiquitously across all human brain regions, with particularly high expression in the cerebellum [42]. RNA-Seq studies demonstrate that Willin/FRMD6 is expressed in neurons, astrocytes, oligodendrocytes, and endothelial cells, with particularly high levels in endothelial cells, and nearly absent in microglia and macrophages in the mouse and human brain [43]. While Willin/FRMD6 immunoreactivity in CNS glial cells has yet to be fully characterized [22], high Willin/FRMD6 immunoreactivity has been found in ependymal cells of the ventricle wall and choroid plexus in mice ([42], http:/www.proteinatlas.org, accessed on 1 August 2021). Willin/FRMD6 immunoreactivity is found in neurons throughout the brain, with particularly abundant staining in the neurons of the olfactory bulb, cerebral cortex, and nuclei of the medulla/pons, and in the cerebellar Purkinje neurons ([42], http:/www.proteinatlas.org, accessed on 1 August 2021). In the diencephalon, Willin/FRMD6 colocalizes with substance P (SP) and arginine vasopressin (AVP) containing fibers in Broca’s diagonal band and the lateral septum; with growth hormone releasing hormone (GHRH) containing fibers in the median eminence [22]. Broca’s diagonal band and the lateral septum, owing to their roles in memory formation and connections to the hippocampus, are thought to play a role in AD pathogenesis [44,45,46,47,48].

In neurons, Willin/FRMD6 is found in the nucleus and the cytoplasm of the soma, the axon, and the synaptosomal compartment. Specifically, Willin/FRMD6 expression is found in the cytoplasm and nucleus of neurons throughout the mouse brain ([42], http:/www.proteinatlas.org, accessed on 1 August 2021). Similarly, Willin/FRMD6 is found in the cytoplasm and nucleus, but not the nucleolus, of mice DRG with three distinct staining patterns: cytoplasmic only, nucleus only, and combined cytoplasmic and nuclear staining [23]. Willin/FRMD6 cytoplasmic staining is vesicular/granular in varicose nerve fibers in the rat CNS and PNS, with younger animals displaying more numerous and intense staining of nerve fibers [22]. Cytoplasmic Willin/FRMD6 granules occasionally move from the neuronal cell body to the axonal transport route [22]. In the spinal cord, Willin/FRMD6 is found in cytoplasmic vesicles at the terminal endings of afferent neurons containing substance P [22]. Willin/FRMD6 is also found in synaptosomal extracts from rat and xenopus brains [22].

Despite these recent findings, our knowledge of Willin/FRMD6′s distribution within the nervous system remains scarce. While several studies have demonstrated the presence and distribution of Willin/FRMD6 within the rat CNS and PNS and the mouse PNS, the distribution of Willin/FRMD6 within the human CNS remains relatively unexplored. Furthermore, what processes, both physiological and pathological, may alter the distribution of Willin/FRMD6 within the nervous system are not yet known. 

## 3. Willin/FRMD6 under Normal Physiological Conditions

### 3.1. Functions of Willin/FRMD6 in Neural Tissues 

As previously mentioned, Willin/FRMD6 was first identified in the rat sciatic nerve [1], where it controls fibroblast migration and proliferation [21]. In this context, Willin/FRMD6 may initiate wound closure in response to nerve injury by inducing fibroblast migration and further downregulation of receptor tyrosine kinase Ephrin B2 and epidermal growth factor receptor [21]. Neuronal response to peripheral nerve injury appears to involve Willin/FRMD6 cytoplasmic-to-nuclear shuttling [23], providing evidence of its differing roles depending on subcellular localization. Such differences in function have been observed for other FERM domain proteins including Merlin, which activates Hippo signaling and recruits Par3 and aPKC to E-cadherin-dependent junctions near the plasma membrane and translocates to the nucleus to directly affect transcription of growth-suppressive genes [49]. Additionally, peripheral nerve injury leads to downregulation of Willin/FRMD6 protein and mRNA expression in small-sized neurons [23]. Notably, these small-sized neurons are DRG nociceptors [23], which may connect to a potential analgesic role of Willin/FRMD6 in the spinal cord [50]. In lesioned motor neurons of the spinal cord, Willin/FRMD6 colocalizes with ATF3 [23], which acts with Jun to repress transcription [51,52,53].

Recently, Willin/FRMD6 has also been shown to play a role in peripheral nerve myelination [37]. Willin/FRMD6 is the target of the cell polarity molecule Crb3 in mouse Schwann cell microvilli [37]. Silencing Willin/FRMD6 in Schwann cells increased myelin internodal length, demonstrating that it plays a role in control of myelin elongation with the effect being more pronounced in thin fibers [37]. Notably, Willin/FRMD6 preferentially labels thin fibers in the cranial nerves [22], suggesting that it may interact with molecules specific to thin nerve fibers to perform its functions.

Willin/FRMD6 may play a role in the function of secretory vesicles and exocytosis in neuronal cells, but the evidence for this remains indirect: Willin/FRMD6 colocalizes with SP-containing vesicles in nerve fibers and in vitro expression of preprotachykinin A, a SP precursor, in pituitary tumor AtT20 and breast cancer MCF-7 cells induces Willin/FRMD6 recruitment to large dense-core vesicles (LDCVs) [22]. LDCVs store and release miRNAs and non-classical neurotransmitters such as neuropeptides and hormones which modulate, rather than induce, synaptic activity [54]. Willin/FRMD6 may function as an adaptor protein for LDCVs in specific neurons, contributing to cell-type specific trafficking and composition of LDCVs [55,56]. Certainly, other FERM domain-containing proteins associate with membrane bound vesicles from the cytoplasmic side and function in regulating their maturation and trafficking by actomyosin remodeling [57,58,59,60,61]. Moreover, Willin/FRMD6 plays a role in processes underlying secretory and synaptic vesicle trafficking and exocytosis such as cortical actin cytoskeleton organization and apical domain size regulation [62,63]. 

### 3.2. Willin/FRMD6 Is an Upstream Regulator of Hippo Signaling

The Hippo signaling cascade involves phosphorylation/activation of the core Hippo kinases MST1/2 and LATS1/2 resulting in phosphorylation and cytoplasmic sequestration/degradation of the transcriptional activators YAP and TAZ in order to control organ size [64], limit cell proliferation [10,63,64], induce apoptosis [21,65], and regulate cell migration and differentiation [66]. While best-known for its role in controlling cell proliferation, Hippo signaling plays important roles in the nervous system, being involved in neuroinflammation, neuronal differentiation, neurodegeneration and neuronal death [67], as well as cell quality control by promoting apoptosis in damaged cells [68].

Upstream regulation of Hippo signaling involves diverse signaling molecules relaying cues related to cell density, mechanical properties of the extracellular environment, actin remodeling, cell polarity, hormones and soluble factors, as well as cellular stress [69]. Initial investigation into Willin/FRMD6′s role as an upstream regulator of Hippo signaling stemmed from studies conducted on the signaling role of the *Drosophila* homologue *Expanded*. However, Willin/FRMD6 lacks the C-terminal domain of *Expanded* that allows it to directly interact with various downstream Hippo signaling components [70]. Nevertheless, Willin/FRMD6 has been shown to be an upstream regulator of Hippo signaling in proliferating mammalian cells including primary rat sciatic nerve fibroblasts [21], MCF10A breast epithelial cells [35], HeLa cells [25], and PC (prostate cancer) cells [71] leading to outputs in cell proliferation, migration, morphology, and mechanotransduction. Overexpression of Willin/FRMD6 in such cells activates Hippo signaling as evidenced by increased phosphorylation of MST1/2, LATS1/2, and YAP/TAZ, as well as increased cytoplasmic retention of YAP [19,23,34]. Willin/FRMD6 knockout in PC cell lines results in decreased phosphorylation of Hippo components including MOB1A, LATS1, and YAP [71]. Transcriptomic, proteomic, and phosphor-proteomic studies further demonstrate that Willin/FRMD6 knockout in PC cells leads to changes in Hippo signaling [71].

Willin/FRMD6′s role in controlling cell proliferation and migration through Hippo signaling is cell-context specific, which may explain why changes in Hippo signaling have been observed with modulating Willin/FRMD6 expression in some studies [21,35] but not others [72]. For example, in fibroblasts, Willin/FRMD6 promotes migration without changes in proliferation [21], while in HeLa cells, Willin/FRMD6 overexpression results in increased migration and decreased cell proliferation [25]. In aging cardiac microvascular endothelial cells (CMECs), Willin/FRMD6 facilitates BDNF-induced migration, as knockdown of Willin/FRMD6 abrogates hallmarks of BDNF-induced migration such as increased actin polarity, stress fiber polymerization, and pseudopod migration [30]. In contrast, Willin/FRMD6 overexpression in neuronal SH-SY5Y cells results in significantly decreased proliferation and migration, but without changes in actin arrangement or cell morphology [73].

Recent studies suggest that Willin/FRMD6 regulation of downstream Hippo components may also be cell-context dependent (Table 1). In contrast to the results in fibroblasts, MCF10A, HeLa, and PC cells, Willin/FRMD6 knockdown in aged CMECS results in increased transcription of MST1/2, LATS1/2, and YAP [30]. In neuronal SH-SY5Y cells, Willin/FRMD6 knockdown leads to decreased nuclear TAZ and decreased YAP expression [73]. Willin/FRMD6 overexpression in SH-SY5Y cells results in significantly increased nuclear TAZ [73]. These results suggest that the mechanism of Willin/FRMD6 regulation of Hippo pathway effectors YAP/TAZ in endothelial and neuronal cells may differ from that in epithelial and fibroblastic cells, where its upstream regulation in mammals was first established [21,35]. Specifically, in epithelial cell lines and fibroblasts, Willin/FRMD6 overexpression leads to decreased nuclear YAP/TAZ, whereas knockdown results in increased nuclear YAP/TAZ. The opposite effect is observed in SH-SY5Y cells [73] and in aged CMECs [30].

### 3.3. ERK, mTOR, and c-Myc Signaling Are Involved in Willin/FRMD6 Functional Output

In addition to Hippo signaling, Willin/FRMD6 is implicated in the regulation of other cellular signaling pathways involved in nutrient-sensing and growth control including ERK, mTOR, and c-Myc. In PC cells, Willin/FRMD6 knockout induces transcriptomic, proteomic, and phosphor-proteomic changes that are associated with modulation of mTOR and c-Myc signaling [71]. Willin/FRMD6 knockout in PC cells results in upregulation of c-Myc signaling components [71]. In glioblastoma (GBM) cells, Willin/FRMD6 does not appear to activate the Hippo pathway, rather it inhibits activation of receptor tyrosine kinases including c-Met and PDGFR and their downstream ERK and AKT kinases [74]. Willin/FRMD6 is also co-localized with c-Met in the nuclei of GBM cells, suggesting that Willin/FRMD6 may negatively regulate c-Met functions such as calcium signaling in the nucleus [74,75]. Crossover between ERK and Hippo signaling is also observed in SH-SY5Y neuronal cells. Willin/FRMD6 overexpression in SH-SY5Y cells results in decreased activation of ERK signaling and decreased expression of transcription factor NeuroD1 [73]. Willin/FRMD6 knockdown in SH-SY5Y cells leads to ERK activation and susceptibility to RA-induced neuronal differentiation as well as increased NeuroD1 expression and nuclear localization [73].

### 3.4. Willin/FRMD6 in Mechanical Signaling and Cell-Cell Junctions

Willin/FRMD6 functions in mechanotransduction by inducing changes in the organization of the actin cytoskeleton. Knockdown of Willin/FRMD6 expression in neuronal SH-SY5Y cells results in alterations in actin organization, evidenced by increased fine membrane extensions, decreased cell area, increased cell elongation, and greater susceptibility to RA-induced differentiation; along with a reduction in the force the cell exerts on its physical environment; the formation of fewer and smaller focal adhesions [73]. These effects are mediated through ERK signaling [73]. Similarly, Willin/FRMD6 knockdown in MCF10A cells [10] and BDNF-treated aged CMECs [30] results in impaired actin organization.

Willin/FRMD6 is also involved in regulating actomyosin contractility at apical junction complexes (AJCs) [3]. Specifically, Willin/FRMD6 recruits aPKC and Par6 to AJCs, where aPKC phosphorylates ROCK, resulting in its exclusion from AJCs and the prevention of excessive contractility [3]. By thus regulating the constriction of the cortical actin cytoskeleton at AJCs, Willin/FRMD6 regulates apical plasma membrane domain size [3], which may be functionally relevant in the nervous system as apical domain size and tension are important in synaptic vesicle exocytosis and endocytosis [62]. Furthermore, the cortical actin cytoskeleton is important in secretory vesicle trafficking and exocytosis and the dynamics of the fusion pore [63].

**Figure 3:**
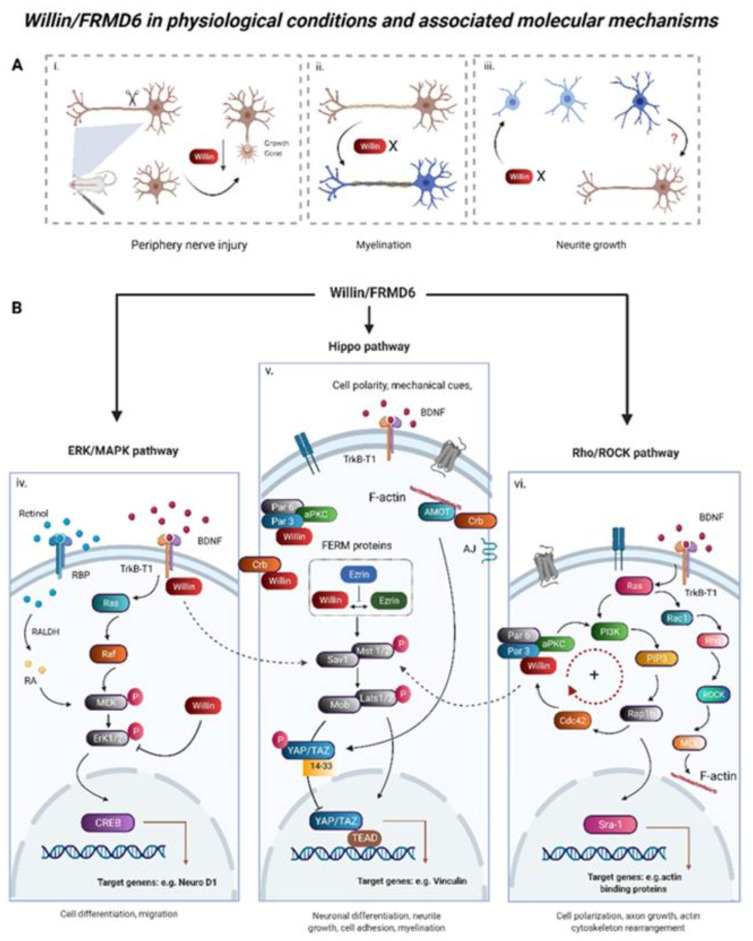
Willin/FRMD6 in physiological conditions and associated molecular mechanisms. Willin/FRMD6 has been implicated in multiple aspects of neuronal function and is associated with several signaling pathways. (**A**) Neuronal functions of Willin/FRMD6 in physiological conditions. (i) Willin/FRMD6 expression is significantly downregulated after mouse peripheral nerve injury by axotomy, indicating a role for Willin/FRMD6 in nerve regeneration. (ii) Willin/FRMD6 silencing leads to increased myelin internodal length in mouse Schwann cells. (iii) Reduction of Willin/FRMD6 promotes neurite growth in SH-SY5Y cells; however, whether Willin/FRMD6 functions similarly in neurons remains to be explored. (**B**) Willin/FRMD6 interconnects with several neuronal differentiation associated signaling pathways. (iv) Willin/FRMD6 knockdown primes SH-SY5Y cells for RA-induced differentiation. Knockdown of Willin/FRMD6 leads to phosphorylation of ERK1/2 in SH-SY5Y cells, indicating activation of the ERK/MAPK signaling pathway (also termed the Ras-Raf-MEK-ERK pathway). In cardiac microvascular endothelial cells, Willin/FRMD6 functions as a downstream effector of BDNF–TrkB-T1 signaling, connecting TrkB-T1 to Hippo signaling. (v) Willin/FRMD6 is an upstream regulator of the Hippo signaling pathway. Canonical Hippo pathway activation involves the phosphorylation of the core Hippo kinases MST1/2 and LATS1/2, resulting in phosphorylation and cytoplasmic sequestration/degradation of terminal effectors YAP/TAZ. In the nucleus, YAP/TAZ bind TEAD transcription factors and initiate pro-survival gene expression. Willin/FRMD6 also interacts with polarity proteins, such as Crb and Par3. (vi) Willin/FRMD6 directly binds to polarity regulator Par3 protein which resides within the PAR/PI3K signaling pathway in epithelial MDCK cells. In neurons, PI3K mediates the production of PIP3 which activates RAP1B and Cdc42 to accumulate at tips of growing axons. The continuously active positive signaling feedback loop can promote rapid growth of axons. Activation of Rho signaling and activation of ROCK can lead to changes in the organization of actin stress fibers. Abbreviations: RA, Retinoic acid; BDNF; Brain-derived neurotrophic factor; RBP, retinol binding protein; RALDH, Retinal dehydrogenase; CREB, cAMP-response element binding protein, Cdc42: cell division cycle 42; PAR 3, partitioning defect 3; PAR 6, partitioning defect 6; aPKC: atypical protein kinase C; RAP1B: Ras-related protein 1b; PIP3, phosphatidylinositol-3,4,5-trisphosphate. Sra-1, Rac1-associated protein. MCL, Myosin regulatory light chain. MDCK cells, Madin-Darby Canine Kidney cells. Created with BioRender.com, accessed on 1 August 2021.

The functions of Willin/FRMD6 are summarized in Figure 3.

## 4. A Link between Willin/FRMD6 and Alzheimer’s Disease

**Figure 4:**
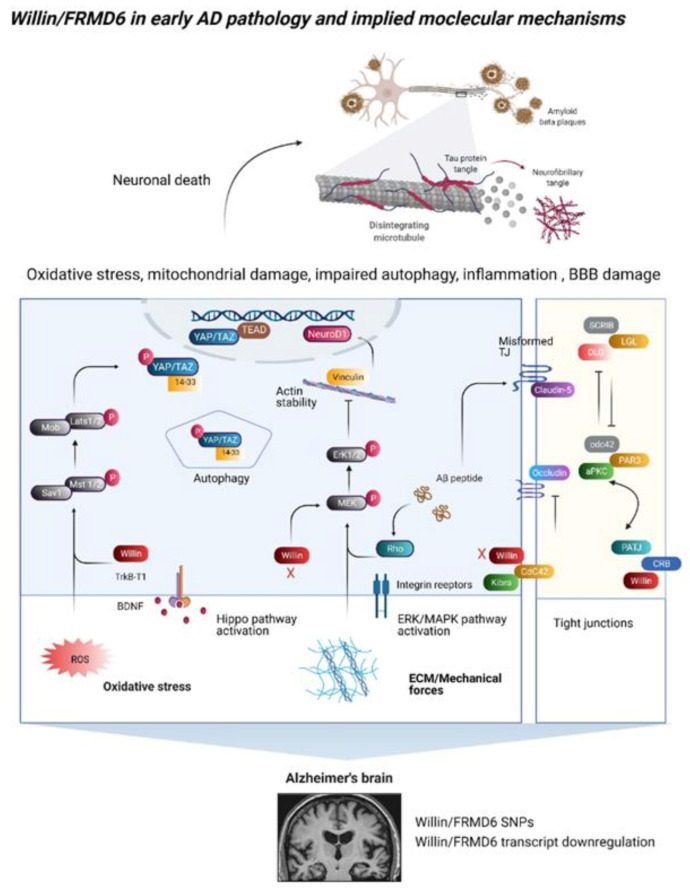
Willin/FRMD6 in early Alzheimer’s disease pathology and implied molecular mechanisms. Mounting evidence indicates a role for Willin/FRMD6 in AD pathogenesis: SNPs in the Willin/FRMD6 gene are associated with differential AD risk; additionally, AD mouse models and AD patient brains show downregulation of Willin/FRMD6 transcripts. Protein and epigenetic modifications in AD Tau and Aβ pathology may also lead to transcriptional and post-transcriptional disruption of Willin/FRMD6 protein levels and function. Alterations in Willin/FRMD6 gene expression has been shown to lead to signaling outputs in the ERK/MAPK, Hippo, mTOR, and c-Myc pathways, as evidenced by phosphorylation of key signaling components. Dysregulation of these pathways is associated with AD-related neuronal death through increased inflammation, impaired autophagy, mitochondrial dysfunction, and oxidative stress, all of which may underly neuronal death in AD. Further consequences of altered Willin/FRMD6 gene expression that may induce neuronal death include changes in actin cytoskeleton organization, which may lead to synaptic dysfunction and defects in axonal mitochondrial trafficking; changes in the extracellular matrix; BBB damage. AD-induced changes in the extracellular matrix may also exacerbate aberrant Willin/FRMD6 mechanotransduction. Through its role in tight junctional complexes, Willin/FRMD6 may be involved in AD pathogenesis by modulating transmembrane protein occludin levels. Aβ oligomers disrupt tight junctions by inducing the redistribution of claudin-5 and ZO-2 to the plasma membrane or inducing RhoA-ROCK signaling activation, causing disruption to tight junction structure and BBB damage. Abbreviations: Scrib module consists of SCRIB, DLG and LGL which is restricted to lateral surfaces. ROS, reactive oxygen species; BBB, blood–brain barrier; Aβ, Amyloid beta; CRB, Crumbs protein; TrkB-T1, Tropomyosin-related receptor kinase type B, T1 isoform. Adapted from ‘Alzheimer’s Brain (Disintegrating Microtubule)’, by BioRender.com (2021). Retrieved from https://app.biorender.com/biorender-templates, accessed on 1 August 2021.

So far, we have seen at both the biochemical and cellular level, Willin/FRMD6 expression can modify pathways and behavior of cells of the nervous system including neuronal cultures, but are these dysregulated in neurological diseases? As explained below, converging areas of interest are now indicating that Willin/FRMD6 may have a role in neurodegeneration (Figure 4).

### 4.1. Single Nucleotide Polymorphisms in the Willin/FRMD6 Gene Are Associated with AD

Several GWAS and imaging studies have highlighted single nucleotide polymorphisms within the promoter and intron regions of the Willin/FRMD6 gene that are associated with AD risk and hippocampal atrophy (Table 2).

Specifically, SNPs within the Willin/FRMD6 gene are associated with AD susceptibility in three separate AD neuroimaging initiative genetics studies [14,15,16] and in a case-control GWAS [13]. The authors highlighted potential Willin/FRMD6 interacting genes underlying its role in neurodegeneration such as SNCAIP, which interacts with alpha-synuclein, and CTBP2, which plays a role in the function of synaptic ribbons [13]. The functional role of Willin/FRMD6 in AD pathogenesis has yet to be established; thus, we will now highlight potential avenues for further exploration based upon known functions and interaction partners of Willin/FRMD6. 

### 4.2. Does Willin/FRMD6 Dysregulation Occur in AD?

There are several avenues of research implying that Willin/FRMD6 dysregulation may occur in AD by both direct but also indirect mechanisms. For example, Willin/FRMD6 mRNA expression is significantly decreased in AD patient brains when comparing total brain mRNA expression [76]. Similarly, AD mouse models (APP NL-G-F/NL-G-F and 3xTg) with both amyloid and tau pathology display significantly decreased cortical expression of Willin/FRMD6 mRNA [77]. Though whether these transcriptional changes translate to protein level changes has yet to be established. However, downregulation of Willin/FRMD6 in neurodegeneration is consistent with observations that Willin/FRMD6 expression decreases with cellular and organismal aging [22].

Further, as indicated below there is evidence that protein and epigenetic modifications in AD may lead to transcriptional and post-transcriptional downregulation of Willin/FRMD6 protein levels and function. Ezrin, which antagonizes Willin/FRMD6′s activation of MST1/2 in HEK-293 cells [35], is upregulated during disease progression in a tauopathy (P301L) mouse model and in AD patients [78]. Moreover, in P301S tauopathy mice, Ezrin expression is specifically upregulated in post synaptic density fractions, with no change in total hippocampal levels [79]. This suggests that AD may induce compartment-specific alterations in protein levels that could phenocopy reduced Willin/FRMD6 expression. MicroRNA miR-454-3p inhibits Willin/FRMD6 protein expression by binding to Willin/FRMD6 mRNA [25] and is strongly upregulated across four independent AD brain tissue datasets [80]. Upregulation of miR-454-3p could thus result in aberrant Willin/FRMD6 protein expression, but this has yet to be directly explored. BDNF mRNA and protein expression are significantly downregulated in AD patient brains [81] and serum [82]. As BDNF has been shown to upregulate Willin/FRMD6 expression, AD-related decreases in BDNF could result in pathological downregulation in Willin/FRMD6. 

Interestingly, FRMD6-AS1 is dysregulated in the AD hippocampus [83,84] and the AD and Huntington’s choroid plexus [85], but whether this antisense RNA is involved in Willin/FRMD6 transcriptional regulation has yet to be explored. However, given the regulatory role of FRMD6-AS2, it is possible that Willin/FRMD6 is also regulated by FRMD6-AS1.

Thus, several lines of evidence point to an association between Willin/FRMD6 downregulation and neuronal death in AD. This may initially seem at odds with the recently discovered functions of Willin/FRMD6 in neuronal SH-SY5Y cells where knockdown in *undifferentiated* cells promotes cell proliferation, migration, neurite growth, and susceptibility to RA-induced differentiation [73]; however, it is important to bear in mind that the functional effects of Willin/FRMD6 are cell-context specific. For example, the effects of altered Willin/FRMD6 on cell proliferation and migration have been shown to vary by cell type [19,28,34,73,74]. Therefore, the functional consequences of Willin/FRMD6 knockdown in a *physiological context* in undifferentiated neuronal SH-SY5Y cells may differ significantly from those in a *pathological context* in differentiated post-mitotic neurons. Certainly, undifferentiated SH-SY5Y cells, which are derived from the neuroblastoma SK-N-SH cell line [86], behave quite differently from mature neurons: SH-SY5Y cells proliferate rapidly, have neuroblast-like morphology with only a few, short processes, and express immature neuronal markers [87], whereas mature neurons are highly polarized, post-mitotic, and have higher energetic demands [88]. Biochemical and biomechanical alterations induced by Willin/FRMD6 knockdown including ERK signaling activation and actin cytoskeleton rearrangement that prime undifferentiated SH-SY5Y cells for RA-induced differentiation may have deleterious consequences for differentiated post-mitotic neurons whose function is highly dependent on the organization of their cytoskeleton. Ultimately, further research needs to be conducted on the function of Willin/FRMD6 in differentiated post-mitotic neurons to fully explain this apparent discrepancy.

Notably, the function of Willin/FRMD6 knockdown in promoting neuronal cell differentiation and neurite growth was observed under physiological rather than pathological conditions. In the AD brain, high levels of Aβ or abnormal tau accumulation along with excessive reactive oxygen species, increased energetic demand, and mitochondrial dysfunction may alter the consequences of Willin/FRMD6 knockdown. In the following section we further detail how the physiological functions and signaling outputs of Willin/FRMD6 downregulation may in turn render cells more vulnerable in the pathological AD milieu. For instance, activation of ERK signaling, which is necessary for inducing neurite growth in SH-SY5Y cells, is associated with increased oxidative stress in AD conditions. Thus, the effects of Willin/FRMD6 downregulation in AD neurons rather than contributing to neurite growth/maintenance may instead exacerbate the deleterious effects of AD including oxidative stress and mitochondrial dysfunction.

## 5. Functional Consequences of AD-Induced Downregulation of Willin/FRMD6

### 5.1. Signaling Outputs in Oxidative Stress, Autophagy, and Neuroinflammation

Modulating Willin/FRMD6 expression leads to signaling outputs in c-Myc, mTOR, ERK, and Hippo pathways along with actin cytoskeletal organization. Through these signaling modules, Willin/FRMD6 may be involved in AD-related neuronal death through oxidative stress, impaired autophagy/mitophagy, and increased inflammation. 

Hyper-activation of Hippo signaling is associated with neuronal death in multiple forms of neurodegeneration including Huntington’s disease [89,90,91], amyotrophic lateral sclerosis (ALS) [92], traumatic brain injury [93], mild cognitive impairment (MCI), and AD [94,95], while inhibition of Hippo pathway activation has been shown to protect against neuronal death [92,93,96]. Increased LATS1 activation is found in cortical neurons of MCI and AD patients, while decreased YAP levels are observed in temporal and occipital tip tissues from AD patients [94]. YAP was one of the key hub genes in a network analysis of AD patient differential gene expression [96]. Activation of Hippo signaling also occurs in AD mouse models as evidenced by elevated levels of c-Abl, p-MST, and p-YAP in the hippocampi of APPswe/PS1dE9 mice [95]. Results from AD mouse models demonstrate that Hippo dysregulation in cortical neurons occurs at an early stage of neuronal death in AD as cytoplasmic sequestration of YAP by intracellular Aβ aggregates and increased LATS1 activation occurs prior to the onset of cognitive impairment in 5xFAD and human mutant APP-KI mouse models [94]. Suppression of early-stage neuronal death by AAV-YAPdeltaC61, a neuron-specific isoform of YAP, reduces later-stage extracellular Aβ and cognitive impairment in these AD mouse models [94]. Notably, in vitro YAP knockdown increases Aβ levels and tau phosphorylation, while overexpression reverses these effects [96]. Whether these changes in Hippo signaling are mediated through dysregulation in Willin/FRMD6 has yet to be explored. Promisingly, since knockdown of Willin/FRMD6 in neuronal cell lines mirrors the effects of hyper-activated Hippo, with increased cytoplasmic TAZ and decreased total YAP levels [73], the effects of decreased Willin/FRMD6 in AD would align with/exacerbate the effects of downstream Hippo activation leading to neuronal death. Moreover, since knockdown of Willin/FRMD6 can itself increase transcription of core Hippo kinases [30], AD-mediated downregulation of Willin/FRMD6 may initiate or exacerbate Hippo dysregulation.

Key pathological consequences of Hippo signaling activation relevant to AD pathogenesis include increased oxidative stress, mitochondrial dysfunction, impaired autophagy/mitophagy, and increased inflammation. Hippo activation in cardiomyocytes disrupts the nuclear interaction between YAP and FOXO1 leading to downregulation of superoxide scavengers catalase and MnSOD, increased oxidative stress, and cell death [97]. Consistently, TAZ knockdown in mouse embryonic fibroblasts results in impaired oxidative phosphorylation, increased oxidative stress, and impaired mitophagy [98]. YAP knockdown in thyroid cancer cells has similar effects including reduced mitochondrial membrane potential, increased mitochondrial permeability transition pore opening, increased ROS production, and increased mitochondrial pro-apoptotic signaling; these effects were exacerbated when combined with MST1 overexpression [99]. Deletion of astrocytic YAP is associated with reactive astrogliosis, microglial activation, and blood–brain barrier dysfunction through increased JAK-STAT inflammatory signaling [100].

Inflammation [101] and oxidative stress [102,103,104,105] both activate MST1, resulting in neuronal and glial cell death, and result from MST1 activation. Activation of MST1 increases ROS production in pancreatic cancer [106], endometriosis, and head and neck squamous cell carcinoma cell lines and results in mitochondrial-driven apoptosis, mitochondrial dysfunction, impaired mitophagy, and excessive mitochondrial fission through elevation of mitochondria fission protein Drp1 [107,108]. MST1 activation also results in impaired autophagy, neuronal apoptosis, and activation of p38-MAPK signaling in motor neurons of a mouse model of ALS [92]. Microglial activation following brain injury is associated with phosphorylation of MST1 leading to increased IκBα and NF-κB signaling resulting in neuroinflammation [109].

While the role of Willin/FRMD6 in Hippo-mediated impairments in oxidative stress, mitochondrial function, autophagy, and neuroinflammation is not yet explored, a potential role for Willin/FRMD6 in these processes is supported by its involvement in mTOR, ERK, and c-Myc signaling pathways. Decreased Willin/FRMD6 expression is associated with activation of c-Myc, mTORC signaling [71], and ERK signaling [73]. Activation of c-Myc is associated with cell cycle re-entry and has been proposed as a mechanism underlying neuronal death [110]. mTOR activation in AD and MCI is linked with inhibition of cellular protein quality control/autophagy and with mitochondrial defects [111]. Similarly, activation of MAP kinase ERK may further exacerbate AD pathology through increased oxidative stress and mitochondrial and neuronal stress [112,113,114]. ERK activation has been associated with oxidative stress and dysfunction in mitochondrial morphology, function, and fission/fusion balance in AD [115].

### 5.2. Changes in the Extracellular Environment

Aging and AD induce significant changes in the CNS microenvironment that disrupt neuronal function through both biochemical and physical mechanisms [116]. Willin/FRMD6, with its recently uncovered role in mediating the mechanical phenotype of neuronal cells, may provide the crucial link between AD and aging associated alterations in the cellular environment and biochemical dysfunction within the cell. AD patient frontal and temporal cortices exhibit increased expression of ECM proteins collagen IV, perlecan, and fibronectin which is positively correlated with Aβ deposition [117]. Furthermore, disruption of ECM proteins has been shown to increase recruitment of circulating leukocytes during inflammation [118]. Willin/FRMD6 has been shown to affect ECM components, as knockout in PC cells results in depletion of ECM and ECM binding components such as collagen, fibronectin, integrin, and laminin [71]. Thus, downregulation of Willin/FRMD6 in AD may partially stem from feedback from AD-induced ECM changes. ECM stiffening may also disrupt Willin/FRMD6-mediated mechanotransduction leading to alterations in downstream signaling pathways.

### 5.3. Blood–Brain Barrier Dysfunction

The blood–brain barrier (BBB) is a specialized membrane composed of endothelial cells that line the cerebral micro vessels and that forms the interface through which neuronal cells communicate with the circulating immune system [119,120,121]. Given that Willin/FRMD6 is highly expressed in brain endothelial cells [43] and in the choroid plexus ([42], http:/www.proteinatlas.org, accessed on 1 August 2021), we briefly discuss potential mechanisms through which Willin/FRMD6 dysfunction may be involved in BBB dysfunction relevant to AD. Knockdown of Willin/FRMD6 [35] in MCF10A cells results in decreased levels of tight junction component occludin. Thus, decreased Willin/FRMD6 in AD may exacerbate Aβ oligomers-induced decreases in expression of ZO-1, claudin-5, and occludin leading to disruption of endothelial cell tight junctions and increased BBB permeability [122,123]. Aβ-induced alterations in tight junctional components are mediated by binding to RAGE and inducing ROS production [122,123,124]. Interaction between Willin/FRMD6 and RAGE in brain endothelial cells presents a potential mechanism underlying BBB dysfunction in AD.

In addition to RAGE-mediated BBB dysfunction, Aβ oligomers also induce RhoA-ROCK signaling/activation to exacerbate BBB damage [125]. Since Willin/FRMD6 is necessary for the recruitment of aPKC to AJCs in order to regulate ROCK activation [3], downregulation of Willin/FRMD6 in AD may decrease the ability of aPKC to inhibit ROCK activation, exacerbating Aβ oligomers-induced BBB damage.

### 5.4. Dysregulation of the Actin Cytoskeleton and Its Dependent Functions

Knockdown of Willin/FRMD6 has also been shown to disrupt actin cytoskeleton organization in epithelial MCF10A [10] and neuronal SH-SY5Y cells [73]. Hence, decreased Willin/FRMD6 levels could exacerbate the deleterious effects of AD on actin cytoskeletal stability, resulting in structural and functional impairment of dendritic spines [126], and of Aβ oligomers on F-actin regulation in dendritic spines, leading to impaired synaptic plasticity [127]. Furthermore, Willin/FRMD6-induced disruption of the actin cytoskeleton could intertwine with the deleterious effects of hyperphosphorylated tau, another biochemical hallmark of AD, on the actin cytoskeleton and on axonal transport along microtubules [128,129]. The actin cytoskeleton, by providing the scaffold for synaptic vesicle formation and mitochondrial trafficking, is also pivotal in the function of synaptic transmission [130,131,132]. As Willin/FRMD6 plays a role in vesicle exocytosis [22] and actin cytoskeleton organization, whether it contributes to synaptic dysfunction in early AD pathogenesis is a promising avenue for future research.

## 6. Willin/FRMD6 Interaction Partners and AD

Recent studies have highlighted several potential protein interaction partners of Willin/FRMD6 that are associated with AD pathogenesis. KIBRA, which activates the Hippo pathway by inducing the phosphorylation of LATS1/2 [133], interacts with Willin/FRMD6 in prostate cancer cells and normal prostate epithelial cell lines [134]. Disruption of the KIBRA-Willin/FRMD6 interaction and Hippo signaling activation occurs in pathological conditions such as prostate cancer [134]. SNPs in KIBRA have been associated with differential memory performance, episodic memory, AD susceptibility, and an age-dependent risk of AD [135,136,137,138,139,140,141]. Furthermore, KIBRA knockout mice display increased neuronal death in the hippocampus [142]. Par3 and aPKC, which interact with Willin/FRMD6 through their JFR domains in MDCK and Ephr4 cells [3], are involved in regulating intracellular trafficking of β-site APP cleaving enzyme 1 (BACE1) and APP in neurons [143,144]. Furthermore, AD patient brains display decreased levels of Par3 [145]. Levels of TrkB-T1, whose intracellular domain interacts with Willin/FRMD6 to recruit it to the plasma membrane in cardiomyocytes [30], are elevated in AD [146]. Furthermore, Aβ decreases expression of the full-length receptor but increases expression of the truncated TrkB-T1 [147].

High-throughput protein–protein interaction screens conducted under the Bioplex project highlight several Willin/FRMD6 interaction partners that have implications in AD pathogenesis [148,149]. Such interaction partners include TP53BP2/ASPP2, which is involved in inflammatory NF-kB signaling and induction of apoptosis through APP-BP1 [150]; FTL, which is involved in iron-binding and has been associated with microglial activation and increased gamma-secretase production in AD [151]; most interestingly, APP, the precursor molecule for Aβ. APP promotes the apoptosis-inducing interaction between MST1 and FOXO3a [152] and forms a transcriptionally active triple protein complex with Mint3 [153] and TAZ/YAP leading to the expression of pro-apoptotic genes [154]. Another FERM-domain containing protein, SNX17, has been shown to be involved in APP trafficking and processing by regulation of APP endocytosis in early endosomes [155]. Nectin-1, which physically interacts with Willin/FRMD6 [34], is proteolytically processed in a manner analogous to that of APP [156]. Interestingly, significantly decreased levels of Par3, which interacts with Willin/FRMD6, are found in AD patients and are associated with impairments in APP trafficking that lead to intracellular accumulation of Aβ [145], potentially through a mechanism involving aPKC and dysregulated BACE1 trafficking in endosomes [144]. These putative interaction partners suggest that a mechanism for Willin/FRMD6′s role in AD could involve regulation of Aβ production and degradation through modulating inflammatory signaling and APP processing.

## 7. Conclusions

As shown here, multiple lines of evidence indicate that Willin/FRMD6 plays an important role in a neuronal context and that it is connected to various mechanisms underlying neurodegeneration, particularly in AD. First, separate GWAS studies suggest a link between Willin/FRMD6 and susceptibility to AD. Second, Willin/FRMD6 is expressed in neurons and its expression and that of its regulatory elements are altered in AD mouse models and AD patients. The role of Willin/FRMD6 in neurodegeneration is further supported by its ubiquitous expression in the nervous system and its physiological functions in neuronal differentiation, myelination, nerve injury repair, and vesicle exocytosis. Additionally, Willin/FRMD6 plays an important role in regulating Hippo, ERK, and mTOR signaling pathways, which are involved in various features of AD pathology including impairment of the blood–brain barrier, oxidative stress, microglia activation, and inflammation. Thus, Willin/FRMD6 presents a promising new target for future AD research and therapeutics.

We propose a model wherein aging is associated with decreased Willin/FRMD6 expression through changes in the biochemical and physical cellular microenvironment. Excessive downregulation occurs with AD resulting in aberrant activation of Hippo signaling in neuronal cells. This also activates signaling pathways associated with hallmarks of AD pathology including oxidative stress, failures in protein quality control and mitochondrial function, and BBB and cytoskeletal dysfunction. These AD-induced dysfunctions themselves exacerbate activation of these signaling pathways, amplifying the deleterious effects. Here, we provided substantial new avenues for research to elucidate the mechanisms underpinning the role of Willin/FRMD6 in AD. Key outstanding questions include:

- Do changes in Willin/FRMD6 transcripts in AD translate to protein level changes?
- Does Willin/FRMD6 function upstream of MST1, LATS1, and YAP/TAZ in the induction of oxidative stress and mitochondrial dysfunction in AD?
- What factors are involved in the control of Willin/FRMD6 signaling function in neuronal cells? Does the subcellular localization of Willin/FRMD6 act as a functional switch between different signaling roles?
- How does Willin/FRMD6 influence Hippo signaling in a neuronal context?
- How does Willin/FRMD6 affect the actin cytoskeleton in neurons and does this contribute to cytoskeleton-dependent functions such as axonal trafficking and synaptic vesicle cycling?
- Are AD-associated decreases in Willin/FRMD6 transcripts associated with a specific subpopulation of neurons? Do these decreases also occur in glial cells?
- Does restoring Willin/FRMD6 expression ameliorate AD-induced perturbations?

## Figures and Tables

**Table 1:** Cell-type specific Wilin/FRMD6 signal transduction.

Pathway	Cell Type	Effect of Willin Overexpression	Effect of Willin Knockdown/Knockout	Reference
Hippo	Primary fibroblasts, MCF10A, HeLa	Activation (increased phosphorylation of MST1/2, LATS1/2, YAP/TAZ; increased cytoplasmic YAP)	Inhibition	[19,23,34]
	PC	Unknown	Inhibition (decreased phosphorylation of MOB1A, LATS1, YAP)	[71]
	Aged CMECs	Unknown	Activation (increased MST1/2, LATS 1/2, YAP transcripts)	[30]
	SH-SY5Y	Possible inhibition (increased nuclear TAZ)	Possible activation (decreased nuclear TAZ, decreased YAP)	[73]
c-MYC	PC	Unknown	Activation	[71]
mTOR	PC	Unknown	Modulation	[71]
ERK	GBM	Inhibition	Unknown	[74]
	SH-SY5Y	Inhibition	Activation	[73]

**Table 2:** Willin/FRMD6 SNPs associated with AD Risk.

SNP	Position	MAF	*p*-Value	SNV	Location	Study
rs7140150	52010799	0.4566	4.77 × 10^−7^	C > T	Intron	[16]
rs11626056	52233276	0.3295	1.18 × 10^−6^	C > A, T	Intron	[15]
rs7153703	51919822	0.213	3.38 × 10^−6^	A > G, T	Promoter	[14]
rs11626565	52075152	0.0599	2.45 × 10^−5^	A > C	Intron	[13]
rs17586545	52035018	0.0334	4.18 × 10^−5^	C > T	Intron	[13]
rs17123958	51942124	0.104	7.59 × 10^−5^	C > G, T	Promoter	[13]
rs12885443	52075653	0.1769	5.34 × 10^−4^	A > C	Intron	[13]

## Data Availability

Not applicable.
